# Sicca syndrome during ipilimumab and nivolumab therapy for metastatic renal cell carcinoma

**DOI:** 10.1002/iju5.12573

**Published:** 2023-01-06

**Authors:** Takuya Segawa, Takanobu Motoshima, Junji Yatsuda, Ryoma Kurahashi, Yumi Fukushima, Yoji Murakami, Takahiro Yamaguchi, Yutaka Sugiyama, Ryoji Yoshida, Hideki Nakayama, Tomomi Kamba

**Affiliations:** ^1^ Department of Urology Kumamoto University Kumamoto Japan; ^2^ Department of Oral and Maxillofacial Surgery Kumamoto University Kumamoto Japan

**Keywords:** immune checkpoint inhibitor, immune‐related adverse event, pilocarpine, sicca syndrome, xerostomia

## Abstract

**Introduction:**

Dry mouth is the main symptom of sicca syndrome, which rarely occurs as an immune‐related adverse event. Here we report a case of sicca syndrome caused by immune checkpoint inhibitor treatment.

**Case presentation:**

A 70‐year‐old man was diagnosed with left renal cell carcinoma after radical left nephrectomy. Nine years later, computed tomography revealed a metastatic nodule in the upper left lung lobe. Subsequently, ipilimumab and nivolumab were administered for recurrent disease. After 13 weeks of treatment, xerostomia and dysgeusia were noted. Salivary gland biopsy revealed lymphocyte and plasma cell infiltration in the salivary glands. Sicca syndrome was diagnosed and pilocarpine hydrochloride was prescribed without corticosteroids, with continuation of immune checkpoint inhibitor therapy. The symptoms alleviated after 36 weeks of treatment, with shrinkage of the metastatic lesions.

**Conclusion:**

We experienced sicca syndrome caused by immune checkpoint inhibitors. Sicca syndrome improved without steroids and the immunotherapy could be continued.

**Abbreviations & Acronyms**
CT: computed tomography
ICI: immune checkpoint inhibitor
irAE: immune‐related adverse event
mRCC: metastatic renal cell carcinoma

**Keynote message**

Sicca syndrome is a rare adverse event of immune checkpoint inhibitor treatment. If a patient complains of dry mouth after treatment with an immune checkpoint inhibitor, sicca syndrome should be considered a differential diagnosis.

## Introduction

Dry mouth and xerostomia are the main symptoms of sicca syndrome, which has a clinical presentation similar to that of Sjogren's syndrome.^1^ Sicca syndrome rarely occurs an irAE.^2^ Here we report a case involving an elderly man who developed sicca syndrome as an adverse event of combination therapy with ipilimumab and nivolumab for mRCC. The patient showed improvement with pilocarpine hydrochloride treatment without discontinuation of immunotherapy.

## Case presentation

**Fig. 1:**
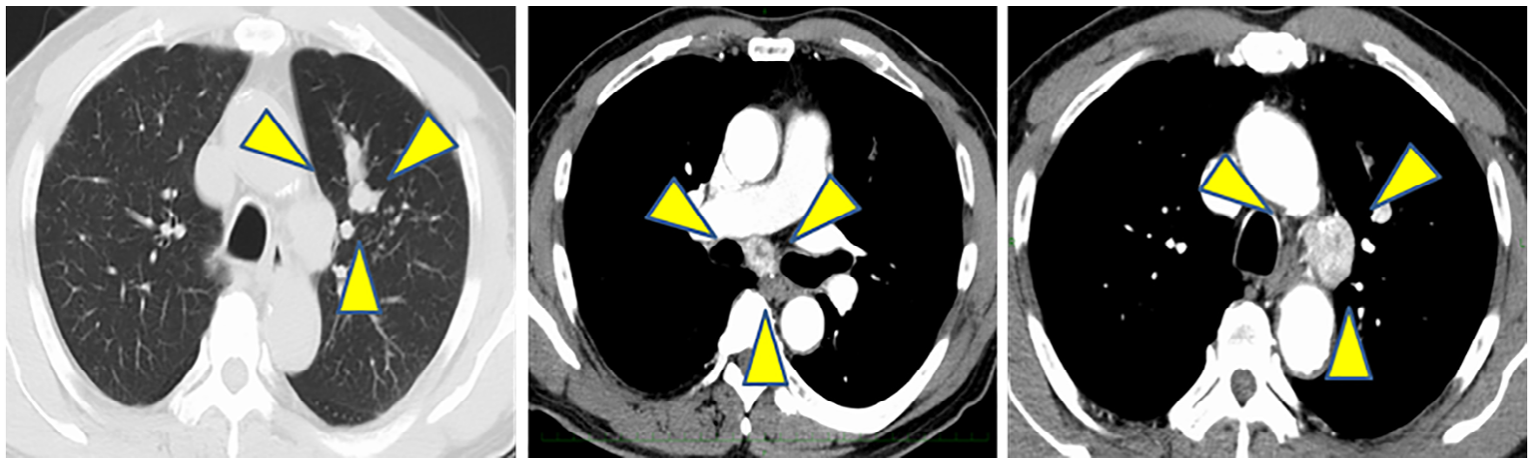
Follow‐up CT findings at 1 year after surgery for mRCC. CT shows a nodule in the upper lobe of the left lung and swelling of the mediastinal lymph nodes.

A 70‐year‐old man was diagnosed with left RCC (clear cell carcinoma, G2, pT1aN0M0) following radical left nephrectomy. Eight years later, he underwent partial lobectomy of the right lung with a diagnosis of primary lung adenocarcinoma (pT2aN0M0). One year after the lung surgery, follow‐up CT revealed a nodule in the upper lobe of the left lung, swelling of the mediastinal lymph nodes (Fig. 1), and a tumor in the right kidney. Transbronchial biopsy from mediastinal lymph nodes was performed to confirm whether the lesions originated from lung cancer or RCC. The pathological diagnosis was RCC metastasis to the lung and mediastinal lymph nodes. Combination therapy with ipilimumab (1 mg/kg) and nivolumab (240 mg/body) was initiated as first‐line therapy because his disease risk was classified as intermediate (hypercalcemia and thrombocytosis) based on the International Metastatic Renal Cell Carcinoma Database Consortium.

**Fig. 2:**
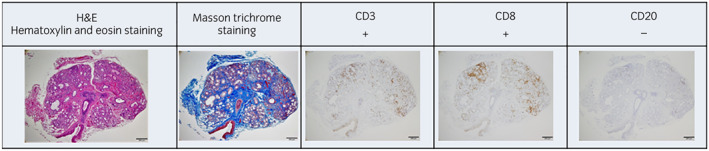
Findings of labial salivary gland biopsy and immunostaining for an elderly man with sicca syndrome caused by ICI treatment for mRCC. Hematoxylin and eosin staining and Masson's trichrome staining show infiltration of lymphocytes and plasma cells in the salivary glands. Immunostaining shows positivity for CD3 and CD8 and negativity for CD20.

**Fig. 3:**
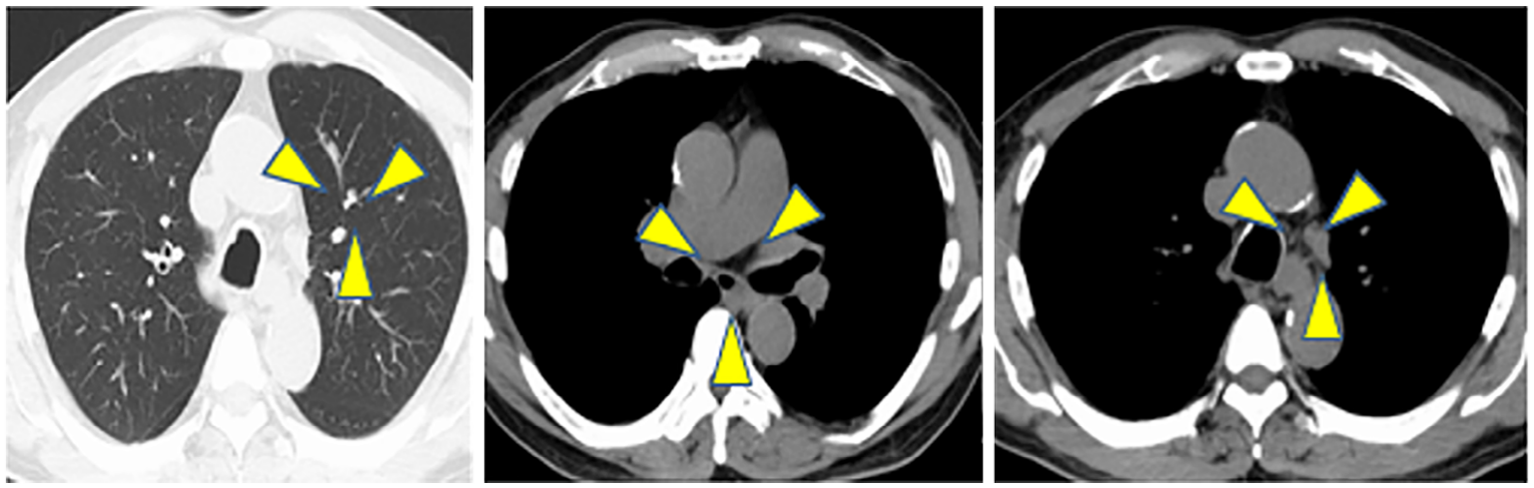
CT findings after 36 weeks of treatment for sicca syndrome caused by ICI treatment for mRCC in an elderly man. CT shows shrinkage of the metastatic lung nodule and mediastinal lymph nodes.

After 13 weeks of treatment, xerostomia and dysgeusia appeared (Common Terminology Criteria for Adverse Events, Grade 2). The Saxon test demonstrated 0.1 g/2 min of saliva, indicating a severe decrease in saliva production. A blood test showed negativity for anti‐nuclear antibody, anti‐SSA antibody, and anti‐SSB antibody. Salivary gland scintigraphy (99mTcO4‐) showed washout of scintillation after lemon juice ingestion. This indicated that the saliva output was maintained while production was reduced. We performed a labial salivary gland biopsy, and the results showed significant infiltration of lymphocytes and plasma cells into the salivary glands. Immunostaining of the salivary glands showed positivity for CD3 and CD8 and negativity for CD20 (Fig. 2). The final diagnosis was sicca syndrome as an irAE caused by ICI therapy. Pilocarpine hydrochloride (15 mg/day) was initiated without corticosteroids, while ICI therapy was continued. After 36 weeks, the result of the Saxon test improved to 5.2 g/2 min, and the patient's symptoms alleviated. At the same time, the metastatic lesions and the tumor in the right kidney showed shrinkage, and an antitumor effect was observed (Fig. 3).

## Discussion

Combination therapy with ipilimumab and nivolumab is an immunotherapy for mRCC. ICIs act on various organs and can cause irAE. Sicca syndrome was first reported by Cappelli *et al*. in 2017.^3^ The reported frequency of occurrence is as low as 0.3%,^2^ and the time to onset is reportedly 1 to 7 months after initiation of ipilimumab and nivolumab.^1^, ^4^, ^5^, ^6^ The clinical features are similar to those of Sjögren's syndrome, with xerostomia, ocular dryness, and low salivary secretion in tests.^1^, ^5^ Blood tests are often negative for antinuclear antibody and anti‐SSA and anti‐SSB antibodies, in contrast to the results in Sjogren's syndrome.^1^, ^5^ In addition, pathological findings of sicca syndrome include CD3^+^ T‐cell infiltration into the salivary glands, whereas in Sjogren's syndrome, inflammatory cells infiltrate the internal ducts of the lobules in the salivary glands, and CD20^+^ B cells form a follicular structure and infiltrate the ductal epithelial structures.^1^, ^5^, ^7^, ^8^, ^9^ In the present case, anti‐nuclear antibody and anti‐SSA and anti‐SSB antibodies were absent, and immunostaining of the salivary glands showed negativity for CD20, which is a B cell lineage marker, and strong positivity for CD3 and CD8, which are T cell lineage markers. These findings suggest that ICI‐activated T cells involved in self‐tolerance may have contributed to the development of sicca syndrome. A correlation between the occurrence of irAEs and therapeutic effects has been reported.^10^, ^11^ In the present case, shrinkage of the lung and mediastinal lymph node lesions and the tumor in the right kidney after ICI treatment was an interesting observation.

With regard to the management of ICI‐associated sicca syndrome, Warner *et al*. reported that the disease almost resolved with either discontinuation of ICI or the use of corticosteroids.^1^ In contrast, Brugués *et al*. reported that there was no need to discontinue ICI treatment, as they found that xerostomia alleviated with basic oral care without the use of corticosteroids in half their patients. The authors proposed an algorithm based on the grade of adverse events.^12^ In the present case, grade 2 xerostomia was treated with oral care and pilocarpine, and it alleviated without the use of corticosteroids or discontinuation of ICI treatment.

## Conclusion

Sicca syndrome caused by ICI therapy is induced by autoimmunity, often with sudden onset, and it is associated with sialadenitis and glandular damage. The incidence of ICI‐related xerostomia may be higher than anticipated, and it is important to consider sicca syndrome as an irAE. We experienced a rare irAE, but was successfully treated without interruption of ICIs or administration of steroids. Oral dryness can decrease the patient's quality of life^13^ and may lead to discontinuation of ICI treatment, resulting in a poor prognosis. Therefore, appropriate management of such irAEs is mandatory to improve the outcomes of patients receiving ICI treatment for cancer.

## Author contributions

**Takuya Segawa:** Writing – original draft. **Takanobu Motoshima:** Writing – review and editing. **Junji Yatsuda:** Writing – review and editing. **Ryoma Kurahashi:** Writing – review and editing. **Yumi Fukushima:** Writing – review and editing. **Yoji Murakami:** Writing – review and editing. **Takahiro Yamaguchi:** Writing – review and editing. **Yutaka Sugiyama:** Writing – review and editing. **Ryoji Yoshida:** Writing – review and editing. **Hideki Nakayama:** Writing – review and editing. **Tomomi Kamba:** Writing – review and editing.

## Conflict of interest

The authors declare no conflict of interest.

## Approval of the research protocol by an Institutional Reviewer Board

Not Applicable.

## Informed consent

A written informed consent was obtained from the patient.

## Registry and the Registration No. of the study/trial

Not Applicable.
